# Independent and combined effects of physical activity and body mass index on the development of Type 2 Diabetes – a meta-analysis of 9 prospective cohort studies

**DOI:** 10.1186/s12966-015-0304-3

**Published:** 2015-12-01

**Authors:** Laura Cloostermans, Wanda Wendel-Vos, Gerda Doornbos, Bethany Howard, Cora Lynn Craig, Mika Kivimäki, Adam G. Tabak, Barbara J. Jefferis, Kimmo Ronkainen, Wendy J. Brown, Susan H. S. J. Picavet, Yoav Ben-Shlomo, Jari Antero Laukkanen, Jussi Kauhanen, Wanda J. E. Bemelmans

**Affiliations:** National Institute for Public Health and the Environment, Bilthoven, The Netherlands; Baker IDI Heart and Diabetes Institute, Melbourne, VIC Australia; Canadian Fitness and Lifestyle Research Institute, Ottawa, Canada; Department of Epidemiology and Public Health, University College London, London, UK; Department of Primary Care and Population Health, University College London, London, United Kingdom; Institute of Public Health and Clinical Nutrition, University of Eastern Finland, Kuopio, Finland; School of Human Movement and Nutrition Sciences, University of Queensland, Brisbane, Australia; Department of Clinical Epidemiology, School of Social and Community Medicine, University of Bristol, Bristol, UK; Department of Internal Medicine, Lapland Central Hospital, Rovaniemi, Finland

**Keywords:** Physical activity, Body mass index, Overweight, Obesity, Meta-analysis

## Abstract

**Background:**

The aim of this harmonized meta-analysis was to examine the independent and combined effects of physical activity and BMI on the incidence of type 2 diabetes.

**Methods:**

Our systematic literature review in 2011 identified 127 potentially relevant prospective studies of which 9 fulfilled the inclusion criteria (total *N* = 117,878, 56.2 % female, mean age = 50.0 years, range = 25–65 years). Measures of baseline physical activity (low, intermediate, high), BMI-category [BMI < 18.4 (underweight), 18.5–24.9 (normal weight), 25.0–29.9 (overweight), 30+ (obese)] and incident type 2 diabetes were harmonized across studies. The associations between physical activity, BMI and incident type 2 diabetes were analyzed using Cox regression with a standardized analysis protocol including adjustments for age, gender, educational level, and smoking. Hazard ratios from individual studies were combined in a random-effects meta-analysis.

**Results:**

Mean follow-up time was 9.1 years. A total of 11,237 incident type 2 diabetes cases were recorded. In mutually adjusted models, being overweight or obese (compared with normal weight) and having low physical activity (compared with high physical activity) were associated with an increased risk of incident type 2 diabetes (hazard ratios 2.33, 95 % CI 1.95–2.78; 6.10, 95 % CI: 4.63–8.04, and 1.23, 95 % CI: 1.09–1.39, respectively). Individuals who were both obese and had low physical activity had 7.4-fold (95 % CI 3.47–15.89) increased risk of type 2 diabetes compared with normal weight, high physically active participants.

**Conclusions:**

This harmonized meta-analysis shows the importance of maintaining a healthy weight and being physically active in diabetes prevention.

**Electronic supplementary material:**

The online version of this article (doi:10.1186/s12966-015-0304-3) contains supplementary material, which is available to authorized users.

## Background

The number of people with diabetes is increasing worldwide. The World Health Organization (WHO) estimates that 285 million people had Type 2 Diabetes (T2D) in 2010, and that this number may increase to 366 million by 2030 [1]. Diabetes is characterized by reductions in insulin production, changes in insulin resistance and glucose uptake. Over time, the increased levels of circulating glucose contribute to the development of cardiovascular and other complications, including blindness and kidney disease. The most common form of diabetes is T2D; at least 85 % of all diabetes cases are T2D [2].

Physical inactivity (PA) and being overweight/obese have frequently been studied as risk factors for T2D. A number of prospective studies have investigated the effect of both PA and overweight (obesity) on incident diabetes [3–8]. Results differed. Rana et al. and Weinstein et al. showed independent associations for both physical activity and overweight/obesity with incident diabetes, with clearly a much larger impact for obesity than for physical activity [7, 8]. Results from Siegel et al. showed risks among those with obesity that were of lesser magnitude [5]. Moreover both Weinstein et al. and Siegel et al. showed only very small, maybe even negligible contributions of physical activity within those categorized as obese [5, 8]. In turn, Manini et al. showed sitting time to be only associated with incident diabetes among obese women [4]. Kriska et al. showed that physical activity contributed to reduced risks of incident diabetes across BMI categories in a high-risk population of Pima Indians, and Hu et al. showed independent associations for both BMI and physical activity among those with a normal, as well as those with impaired, glucose tolerance [3, 6]. Although these studies contribute to the understanding of this subject, they were limited to a certain extent. Study populations tended to be unbalanced in that some included only one of both sexes [4, 5, 7, 8] or a high risk group [3, 6]. In some cases, either or both BMI and physical activity were analyzed dichotomously [5, 8, 9], instead of using a wider range of categories.

Because of the heterogeneity in methods, meta-analysis of published results from cohort studies will not result in more uniform conclusions. The InterAct-consortium was the first to publish the results of a meta-analyses beyond published results, using standardized data from a case-cohort study nested within a large pan-European study (*N* = 27,364 including 11,230 incident type 2 diabetes cases). Physical activity was assessed using three questions from which a four-category index was derived, and BMI was calculated using a combination of self-reported and measured values for height and weight. The conclusion was that among both men and women, higher PA levels are associated with reduced risk of developing T2D, independent of BMI [10].

In this paper we build on the standard protocols for conducting meta-analyses, which involve conducting a systematic review of the literature followed by a meta-regression using published results from the included studies. Instead, in this paper, we first describe the methods used to identify existing eligible prospective cohort studies, and then how we asked researchers to re-analyze their data according to a series of predefined and harmonized proportional hazards models, so that the results could be included in our harmonized meta-regression. The aim was to examine both the independent and combined effects of PA and BMI on the development of T2D.

In contrast with the Interact study which assessed physical activity across BMI-categories, our harmonized analysis adds to understanding of the combined role of physical inactivity and high BMI to the development of T2D, using data from cohort studies of populations in and outside Europe.

## Methods

### Identifying eligible cohorts

A Medline search was conducted in September 2011 to identify prospective cohort studies that included data on the associations between both PA and BMI with T2D incidence. Cohort studies published between January 1989 and September 2011 were selected. The following search terms were used: Physical activity (‘physical activit*‘, ‘physically active lifestyle*‘, ‘vigorous activit*‘, ‘motor activity‘, ‘exercise‘,‘leisure and activit*‘, ‘recreation*‘, ‘pedestrian*‘, ‘walking‘, ‘running‘, ‘jogging‘, ‘bicycling‘, ‘cycling‘, ‘skating‘, ‘sport‘, ‘sports‘, ‘sporting‘, ‘fitness‘, ‘active‘, ‘commuting‘, ‘commuting activity‘, ‘active transport‘, ‘travel behavio?r‘, ‘inactivity‘, ‘sedentary behavio?r‘, ‘television‘, ‘tv‘, ‘personal computer*‘, ‘pc’) and diabetes (‘diabetes‘, ‘diabetic‘,‘niddm‘, ‘iddm´). To identify additional cohorts of interest, reference lists and reviews of the original publications were checked and we asked experts to review and supplement this list to ensure no potential cohort had been missed. This resulted in 127 cohorts that could potentially provide information about PA, BMI and T2D incidence.

**Fig. 1:**
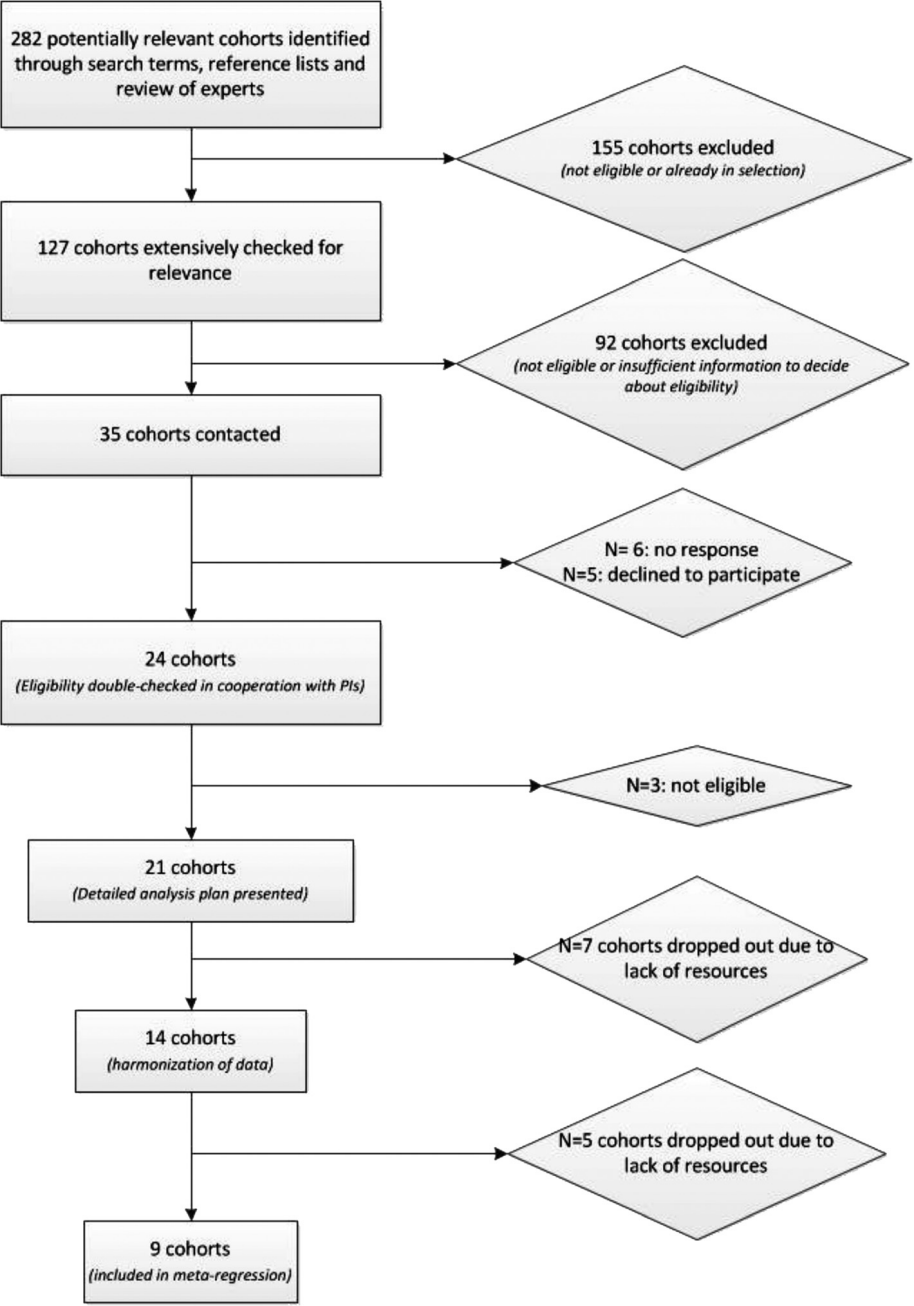
Flow chart showing the selection of the cohorts

Cross-sectional, case–control studies, clinical trials and other intervention studies which aimed to reduce weight and/or increase physical activity, to reduce T2D incidence were excluded. We included cohort studies according to their study characteristics rather than on the basis of the published analyses. Studies were included if: they included a generally ‘healthy’, predominantly white (>50 %) sample; the age range was 25–65 years at baseline; the study included measures of PA (with an indication of frequency, duration and intensity), height and weight, educational level or socio-economic status and smoking; the follow-up was at least 4 years; and incidence T2D was available at follow-up. Applying these criteria reduced the number of 127 potential cohorts to 35 eligible cohorts (Fig. 1).

### Selecting and contacting eligible cohorts

The principal investigators (PIs) of the 35 cohorts were contacted to invite them to participate in this harmonized meta-analysis, after verifying the eligibility of their cohort. Reimbursements of costs or financial incentives were not provided. PIs were offered the possibility to delegate statistical analyses to RIVM.

Six cohorts did not respond and five cohorts declined to participate (*N* = 3 not interested, *N* = 2 without reason). For 24 cohorts eligibility was double-checked in cooperation with the PIs. Three additional cohorts proved not to be eligible and thus were excluded. The detailed analysis plan was presented to 21 cohorts. Lack of resources (*N* = 4 lack of resources at PIs offices, *N* = 3 obligatory payments to be made by the leading authors of this paper) resulted in the exclusion of seven additional cohorts. We compared the detailed study designs of the 14 remaining studies and finalized an analysis plan that ensured harmonized measurement of variables and analysis for all cohorts. This whole process of contacting cohorts, double-checking eligibility and harmonizing measurements proved to be very intensive and time consuming. During this process that took approximately one and a half year, five cohorts dropped out. The meta-analysis presented in this paper included data from nine cohorts, all of which were approved by local ethical committees and were carried out in compliance with the Helsinki declaration.

### Data preparation

In the analysis plan we specified the definition, measurement and cut-off values of variables. The goal was for each study to comply exactly, or as closely as possible with the following definitions of the explanatory and outcome variables.

#### Physical activity

Physical activity measures (frequency, intensity, and duration) for a large range of activities (see Additional file 1: Table A.1) were available. Our measures are based on two domains of activity:

- leisure time physical activities (including walking, gardening, shopping and home maintenance)
- active commuting

The measures exclude activities at work or household chores. Minutes per week spent in low, medium and high physical activity were categorized as shown in Table 1. Cut off values correspond with Global Physical Activity Guidelines [11].

**Table 1:** Definition of Physical activity (PA) categories

Low PA	:	Sum of PA-minutes/week = 0 min
Medium PA	:	0 min < Sum of PA-minutes/week < 150 min
High PA	:	Sum of PA-minutes/week ≥ 150 min

#### Body mass index (BMI)

BMI was calculated, as weight (kg) divided by height squared (m^2^). Four BMI categories were defined, according to the WHO standard WHO [12]: underweight (BMI < 18.5 kg/m^2^), normal weight (18.5 ≤ BMI <25 kg/m^2^), overweight (25 ≤ BMI <30 kg/m^2^) and obese (BMI ≥30 kg/m^2^). Height and weight were self-reported in two studies and measured at baseline examination in the eight other studies (see Additional file 1: Table A.1).

#### Diabetes Type 2 (T2D) incidence

Incidence of T2D was measured within a follow-up period of (or as close as possible to) 10 years after baseline. The date of diagnosis for incident cases was recorded as the date for onset of diabetes reported in a follow-up questionnaire or at a follow-up examination. If year was all that was available, the diagnosis date was set to July 1^st^ of that year. If date of diagnosis was not available, the midpoint of a time-period between examinations within which diabetes was first reported was taken.

According to clinical guidelines, a diagnosis of T2D should preferably be based on measured plasma glucose levels [13]: fasting plasma glucose (≥7.0 mmol/l), non-fasting plasma glucose (≥11.1 mmol/l), 2-h post-load plasma glucose (≥11.0 mmol/l) or HbA1C (>6.5 %). Alternatively, whole blood glucose levels can be used (fasting glucose: ≥ 6.1 mmol/l, non-fasting glucose: ≥11.1 mmol/l, 2-h post-load glucose: ≥11.1 mmol/l). Three studies had measured glucose levels. The remaining six studies used self-reported questionnaire items on diabetes such as: ‘Has a doctor told you, you have diabetes?’ or ‘Do you have type 2 diabetes?’) [13]. See Additional file 1: Table A.1 for details on assessment of T2D in each study.

#### Covariates

Participants’ age (in years), gender, educational level and smoking status were assessed at baseline. *Educational level* was categorized as: low (intermediate secondary school or less), medium (intermediate vocational or higher secondary education) or high (higher vocational education or university). *Smoking* was categorized as: non-smoker, ex-smoker or current smoker.

#### Selection of study population in each cohort

Only participants aged 25–65 years were included. Pregnant women, and cases with Type 1 diabetes (T1D) or T2D at baseline (prevalent cases) were excluded. Furthermore, cases with missing values on one of the following characteristics: age, gender, educational level, smoking status, physical activity, body mass index or T2D-event were excluded. For each cohort a descriptive report on the characteristics of the selected study population was compiled.

#### Longitudinal analyses of individual cohort data

The analysis plan included a protocol with instructions and example code in SAS for Cox-regression analyses (Table 2).

**Table 2:** Overview of analyzed relationships (Cox regression analyses) and requested output

Relation	Model 1 (no adjustment)	Model 2 (adjustment for covariates)	Requested output
Physical activity (PA) on T2D	PA-categories:	Age, Gender, Educational level, Smoking, BMI-categories	HRs, Betas, SEs and 95 % Confidence intervals
	Low PA		
	Medium PA		
	High PA^a^		
Body Mass Index (BMI) on T2D	BMI-categories:	Age, Gender, Educational level, Smoking, PA-categories	HRs, Betas, SEs and 95 % Confidence intervals
	Underweight		
	Normal weight^a^		
	Overweight		
	Obesity		
Combined effect of Physical activity (PA) and Body Mass Index (BMI) on T2D	Combined categories:	Age, Gender, Educational level, Smoking	HRs, Betas, SEs and 95 % Confidence intervals
	High PA – Normal weight^a^		
	Low PA – Underweight		
	Low PA – Normal weight		
	Low PA – Overweight		
	Low PA – Obesity		
	Medium PA – Underweight		
	Medium PA – Normal weight		
	Medium PA – Overweight		
	Medium PA – Obesity		
	High PA – Underweight		
	High PA – Overweight		
	High PA – Obesity		

^a^Reference category

Data from six studies were analysed by their primary investigators, and in the remaining three studies the original study data were analyzed at the National Institute for Public Health and the Environment (RIVM). Analyses were done with SAS 9.2 or STATA 12. Table 2 presents an overview of the Cox-regression analyses requested for the associations between physical activity and BMI and T2D. Each model was run first without covariates and then with covariates (age, gender, educational level, smoking).

When each cohort’s regression results were received at RIVM, checks were performed to verify the definition of variables and that analyses had been conducted according to the protocol. This entailed extensive communication between the PIs of each study and the first author of this paper. Results were returned on average 4 times before they were fully compliant with the protocol.

In the subset of studies for which original data were analyzed at RIVM, the proportional hazard assumption for the explanatory variables in Cox regression model was tested [14].

#### Meta survival regression

Because of differences within the 9 study populations, a random effects model was used [15]. Heterogeneity was tested with a likelihood ratio test. Hazard ratios and standard errors were pooled using the restricted maximum likelihood method.

Summary estimates for categories of physical activity with reference level high physical activity and categories of BMI with reference level normal weight, were calculated. These models were unadjusted or adjusted for age, gender, educational level, and smoking. Physical activity models were additionally adjusted for BMI and BMI models were additionally adjusted for physical activity.

## Results

Descriptive statistics of the nine included studies are presented in Table 3. *N* = 117,878 participants were included; individual studies varied in size from 1087 (KIHD) to 86,368 (CPS-II), the weight of the latter was substantial in the pooled analysis, being much larger than other studies. Three studies consisted entirely of male participants (BHRS [16], CaPS [17], KIHD) and one study consisted entirely of female participants (ALSWH [18]). Mean follow-up time was 9.1 years. A total of 11,237 incident T2D cases were recorded. The mean time to T2D event ranged from 4.2 years (KIHD) to 8.8 years (PALS [19]).

**Table 3:** Descriptive statistics of 9 individual cohort studies included in the meta-analysis, *n* = 119,396 participants

	Cohort	Country	*N*	Mean age	% male	Mean time to T2D event (Years)	% Incident cases T2D (Absolute number)	PA categories % (Absolute number)	BMI categories % (Absolute number)	Mean BMI
1	Australian Longitudinal Study on Women’s Health (ALSWH) [18]	Australia	9599	49.6	0 %	4.9	4.6 % (441)	Low = 17.7 % (1695)	underweight = 1.6 % (140)	26.2
								Med = 25.8 % (2474)	normal weight = 47.8 % (4592)	
								High = 56.6 % (5430)	mod overweight = 31.6 % (3036)	
									obese = 19.1 % (1831)	
2	The Australian Diabetes, Obesity and Lifestyle study (AusDiab) [24]	Australia	4327	47.1	46 %	4.3	3.0 % (130)	Low = 39.6 % (1712)	underweight = 0.8 % (35)	26.5
								Med = 57.8 % (2503)	normal weight = 40.4 % (1748)	
								High = 2.6 % (112)	mod overweight = 39.5 % (1709)	
									obese = 19.3 % (835)	
3	British Regional Heart Study (BRHS) [16]	United Kingdom	4720	49.3	100 %	7.0	1.7 % (82)	Low = 31.3 % (2547)	underweight = 0.4 % (19)	25.4
								Med = 14.8 % (698)	normal weight = 46.4 % (2188)	
								High = 54.0 % (2547)	mod overweight = 46.3 % (2184)	
									obese = 7.0 % (329)	
4	Caerphilly Prospective Study (CaPS) [17]	United Kingdom	2034	56.6	100 %	4.7	4.9 % (99)	Low = 0 % (0)	underweight = 1.2 % (25)	26.4
								Med = 18.6 % (378)	normal weight = 32.5 % (660)	
								High = 81.4 % (1656)	mod overweight = 52.4 % (1065)	
									obese = 14.0 % (284)	
5	Cancer prevention study-II Nutrition Cohort (CPS-II)	United States	86368	58.7	42 %	6.3	11.5 % (9948)	Low = 1.3 % (1119)	underweight = 1.1 % (969)	26.1
								Med = 30.3 % (26166)	normal weight = 43.8 % (37851)	
								High = 68.4 % (59083)	mod overweight = 39.5 % (34123)	
									obese = 15.5 % (13425)	
6	Doetinchem Cohort Study [25]	The Netherlands	3401	46.7	44 %	5.6	2.0 % (67)	Low =0.7 % (24)	underweight = 0.8 % (27)	25.6
								Med = 2.1 % (71)	normal weight = 47.2 % (1605)	
								High = 97 % (3306)	mod overweight = 40.7 % (1383)	
									obese = 11.4 % (386)	
7	Kuopio Ischaemic Heart Disease Risk Factor Study (KIHD)	Finland	1087	51.7	100 %	4.2	5.1 % (55)	Low = 0.6 % (6)	underweight = 0.1 % (1)	26.8
								Med = 11.4 % (124)	normal weight = 34.4 % (374)	
								High = 88 % (957)	mod overweight = 50.4 % (548)	
									obese = 15.1 % (164)	
8	Physical Activity Longitudinal Study (PALS) [19]	Canada	1487	41.0	48 %	8.8	2.2 % (32)	Low = 2.1 % (31)	underweight = 2.2 % (32)	25.2
								Med = 30 % (446)	normal weight = 50.7 % (754)	
								High = 67.9 % (1010)	mod overweight = 36.3 % (540)	
									obese = 10.8 % (161)	
9	Whitehall-II study (WH-II) [26]	United Kingdom	4855	49.1	70 %	8.5	7.9 % (383)	Low = 0 % (0)	underweight = 0.95 (46)	25.3
								Med = 10 % (508)	normal weight = 51.99 (2524)	
								High = 89.5 % (4347)	mod overweight = 38.00 (1845)	
									obese = 9.06 (440)	

The assumption of proportional hazards was not met for the lowest category of BMI, underweight (*P* < 0.001). Since numbers in this category were small and because of the non-proportionality of hazards, underweight was not included in the results.

The results of the meta-analysis showing the relationships between physical activity and T2D are shown in Table 4 (also see Additional file 1: Figure B1 and B2). In the unadjusted model, the risk of T2D was 64 % higher in the low PA category compared with the high PA category. Adjustment for confounders attenuated this estimate to 1.23 (95 % CI: 1.09–1.39). For medium PA risk estimates were also attenuated, but to a lesser degree.

**Table 4:** Meta- analysis of baseline physical activity categories as determinant of T2D development

Physical activity on T2D		Model 1^a^		Model 2^b^		
	*N*	HR	95 % CI	HR	95 % CI	Heterogeneity *p*-value
High physical activity	9	1.00		1.00		
Medium physical activity	9	1.11	0.86–1.43	1.08	1.04–1.13	1^d^
Low physical activity	7^c^	1.64	1.45–1.85	1.23	1.09–1.39	1^d^

^a^Model 1: T2D = physical activity level categories

^b^Model 2: Model 1 + age + gender + educational level + smoking + body mass index categories

^c^No estimates for CaPS and WH-II

^d^Between study variance is estimated as zero

Relationships between baseline BMI and risk of developing T2D are shown in Table 5 (also see Additional file 1: Figure B3 and B4) . The associations between being overweight or obese and risk of T2D were stronger than the associations of lower levels of physical activity and T2D. The summary hazard ratios for developing T2D in participants who were overweight or obese were 2.33 (95 % CI: 1.95–2.78) and 6.10 (95 % CI: 4.63–8.04) respectively.

**Table 5:** Meta-analysis of baseline body mass index categories as determinants of T2D development

Body mass index on T2D		Model 1^a^		Model 2^b^		
	*N*	HR	95 % CI	HR	95 % CI	Heterogeneity *p*-value
Normal weight	9	1.00		1.00		
Overweight	9	2.46	2.07–2.91	2.33	1.95–2.78	0.096
Obesity	9	6.68	4.94–9.03	6.10	4.63–8.04	<.0001

^a^Model 1: T2D = BMI categories

^b^Model 2: Model 1 + age + gender + educational level + smoking + physical activity level categories

**Fig. 2:**
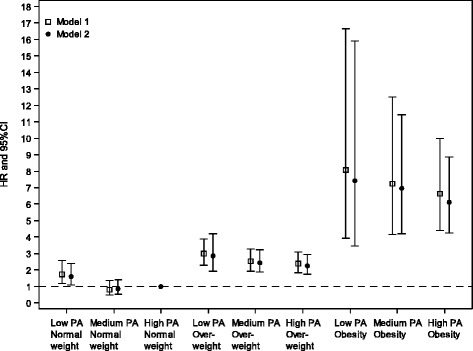
Meta-analysis of combined classes of baseline physical activity and body mass index on T2D development. Model 1: T2D = combined physical activity level and BMI categories. Model 2: Model 1 + age + gender + educational level + smoking

The combined effect of physical activity and body mass index levels on T2D was assessed by creating separate indicators for combinations of PA and BMI. Results are in Table 6 and graphically shown in Fig. 2.

**Table 6:** Meta-analysis of combined classes of baseline physical activity (PA) and baseline body mass index on development of T2D

Combined PA x BMI classes		Model 1^a^	Model 2^b^
	*N*	HR (95 % CI)	HR (95 % CI)
High PA, normal weight		1.00	1.00
Medium PA, normal weight	9	0.80 (0.46–1.38)	0.84 (0.50–1.39)
Low PA, normal weight	6	1.75 (1.19–2.58)	1.61 (1.09–2.37)
High PA, overweight	9	2.38 (1.83–3.10)	2.26 (1.74–2.93)
Medium PA, overweight	9	2.52 (1.94–3.28)	2.45 (1.87–3.20)
Low PA, overweight	7	3.00 (2.31–3.88)	2.86 (1.93–4.22)
High PA, obese	9	6.62 (4.39–10.00)	6.13 (4.25–8.84)
Medium PA, obese	9	7.21 (4.16–12.51)	6.93 (4.20–11.43)
Low PA, obese	7	8.07 (3.91–16.67)	7.43 (3.47–15.89)

^a^Model 1: T2D = combined physical activity level and BMI categories

^b^Model 2: Model 1 + age + gender + educational level + smoking

The single reference group in this analysis was ‘high physical activity/normal weight’. The highest hazard ratio was among individuals who were both obese and had low physical activity (HR 7.43, 95 % CI 3.47–15.89). Except for ‘medium physical activity/normal weight’, hazard ratios were higher for all other combinations of PA and BMI than in the comparison group ‘high physical activity/normal weight’ (significance level *p* <0.05). Within the overweight and obese BMI categories, the summary hazard ratios of T2D decreased with higher levels of physical activity. The p value for the test of linear trend across the three overweight combinations (low PA/overweight, medium PA/overweight, high PA/overweight) in the adjusted model was *p* = 0.0076 and across the three obese combinations (low PA/obese, medium PA/obese, high PA/obese) in the adjusted model : *p* = 0.5780.

## Discussion

In contrast with previous studies which have assessed physical activity across BMI-categories, our harmonized analysis adds to understanding of the combined role of physical inactivity and high BMI in the development of T2D, using data from cohort studies of population samples from within and outside Europe. In this meta-analysis of harmonized data from 9 prospective cohort studies - in mutually adjusted models - being overweight, obese (compared to normal weight) and having low physical activity (compared to high physical activity) were associated with an increased risk of incident type 2 diabetes (hazard ratios 2.33, 95 % CI 1.95–2.78; 6.10, 95 % CI: 4.63–8.04, and 1.23, 95 % CI: 1.09–1.39, respectively). Individuals who were both obese and had low physical activity had a 7.4-fold (95 % CI 3.47–15.89) increased risk of type 2 diabetes, compared with normal weight, high physically active participants.

This study showed slightly weaker individual associations between physical activity, overweight and obesity and diabetes than previous studies have shown. For example, Jeon et al. calculated a summary relative risk based on 10 prospective cohort studies of 1.45 (95 % CI: 1.20–1.72) for being sedentary compared to regular participation in moderate physical activity [20]. Aune et al. calculated summary relative risks for several physical activity measures based on a mix of prospective cohort, case-cohort, nested case–control studies and randomized trials. In their analysis for total physical activity, 14 prospective cohort studies were included. The summary estimate for low versus high total physical activity was 1.54 (95 % CI: 1.41–1.69). For leisure time physical activity they calculated a summary estimate of 1.35 (95 % CI: 1.27–1.41) based on 55 prospective cohort studies [21]. The stronger association in the Jeon et al. study may be explained by the fact that they included a large range of physical activity domains. In contrast, our PA-measure was based on only two domains of physical activity: leisure time physical activity and transport-related physical activity (specifically active commuting). Aune et al. showed a weaker association based on leisure time physical activity alone than for total physical activity. Our summary estimate for low versus high physical activity was 1.23 (95 % CI: 1.09–1.39). Abdullah et al. calculated a summary relative risk based on 18 prospective cohort studies of 7.28 (95 % CI: 6.47–8.28) for obesity and 2.92 (95 % CI: 2.57–3.32) for overweight, compared with normal weight [22]. Our summary estimates were 6.10 (95 % CI: 4.63–8.04) and 2.33 (95 % CI: 1.95–2.78) respectively.

Furthermore we observed stronger associations with T2D for obesity than for physical activity. This is in line with previous studies. In a systematic review, Fogelholm found that in six out of eight included studies the combination of being obese and physically active was a greater health hazard in terms of T2D than having normal weight and being inactive [23]. The InterAct-consortium concluded that for both men and women lower levels of physical activity were associated with an increased risk of T2D across BMI-categories [10]. Similarly, our findings showed decreasing HRs for T2D incidence within the overweight and obese BMI categories for increasing levels of physical activity. This trend was statistically significant among those categorized as overweight (p for trend: 0.0076) but not for those categorized as obese (p for trend: 0.5780). In future studies, using objective measures for physical activity, as well as height and weight, could help to further assess the relative importance of adiposity and physical activity in relation to T2D.

The strength of our study is that results are not based on published results, but on (re) analysis of data from individual cohorts, following a standardized protocol. This harmonized analysis reduces the heterogeneity of the study results. As the studies were not published, we could not assess publication bias in the standard way. However, there was potential for bias because data from only nine of the 21 eligible prospective cohort studies could be included. As the cohorts that were not included have not published on the combined effect of physical activity and BMI on T2D, it is difficult to judge the direction or the extent of bias in our summary estimates. Despite our efforts to standardize, not all differences between cohorts could be resolved, because the PA questionnaires and methods of T2D case ascertainment differed across cohorts. The quality of measurement of overweight and obesity was likely to have been higher than for PA, which was measured using quite crude methods in some studies. This would have affected the effect sizes, and may have consequences for interpreting the relative importance of both risk factors in T2D development. Use of objective measures of PA in future studies will improve these limitations.

## Conclusions

Our study demonstrates important associations between increasing levels of BMI and decreasing levels of physical activity on the development of T2D. Participants who were obese and who had the lowest levels of physical activity were most at risk for developing T2D. Our findings underline the importance of maintaining a healthy weight and also of being physically active in order to reduce risk of developing T2D.

## Additional file

Additional file 1: Table A.1 – Characteristics of cohort studies included in the meta-analysis. **Figure B.1**. Hazard ratios and 95 % CI of baseline low physical activity on development of T2D. Model is adjusted for age, gender, educational level, smoking and BMI. **Figure B.2**. Hazard ratios and 95 % CI of baseline medium physical activity on development of T2D. Model is adjusted for age, gender, educational level, smoking and BMI. **Figure B.3**. Hazard ratios and 95 % CI of baseline overweight on development of T2D. Model is adjusted for age, gender, educational level, smoking and physical activity. **Figure B.4**. Hazard ratios and 95 % CI of baseline obesity on development of T2D. Model is adjusted for age, gender, educational level, smoking and physical activity. (DOCX 39 kb)
