# Supplementary material for: Independent and combined effects of physical activity and body mass index on the development of Type 2 Diabetes – a meta-analysis of 9 prospective cohort studies
Source: Int J Behav Nutr Phys Act. 2015 Dec 1;12:147. doi: 10.1186/s12966-015-0304-3 (PMC4666059; doi:10.1186/s12966-015-0304-3)
Supplement: Additional file 1: Table A.1 — – Characteristics of cohort studies included in the meta-analysis. Figure B.1. Hazard ratios and 95 % CI of baseline low physical activity on development of T2D. Model is adjusted for age, gender, educational level, smoking and BMI. Figure B.2. Hazard ratios and 95 % CI of baseline medium physical activity on development of T2D. Model is adjusted for age, gender, educational level, smoking and BMI. Figure B.3. Hazard ratios and 95 % CI of baseline overweight on development of T2D. Model is adjusted for age, gender, educational level, smoking and physical activity. Figure B.4. Hazard ratios and 95 % CI of baseline obesity on development of T2D. Model is adjusted for age, gender, educational level, smoking and physical activity. (DOCX 39 kb) [file 12966_2015_304_MOESM1_ESM.docx]

**Additional file 1**

**APPENDIX A - Table A.1– Characteristics of cohort studies included in the meta-analysis**

|  | **Cohort** | **Country** | **PHYSICAL ACTIVITY**  **activities included in questionnaire** | **Possibility to calculate**  **MET-minutes** | **BMI** | **Physical fitness** | **Additional Confounders** | **T2D** |
| --- | --- | --- | --- | --- | --- | --- | --- | --- |
| 1 | Australian Longitudinal Study on Women’s Health (ALSWH) | Australia | - Walking  - Moderate activity in the last week  - Vigorous activity in the last week | Yes | Self-report | No | - - Hypertension - - Alcohol | Self-report |
| 2. | The Australian Diabetes, Obesity and Lifestyle study (AusDiab) | Australia | - Walking - Gardening/heavy work around the yard - Vigorous PA - Moderate PA | Yes | Exam | No | - - Hypertension - - Alcohol - - Cholesterol   - Family history T2D | Plasma values |
| 3. | British Regional Heart Study (BRHS) | United Kingdom | - Walking - Cycling - Activity in weekends - Active PA | No | Exam | No | - Alcohol  - Hypertension | Self-report |
| 4. | Caerphilly Prospective Study | United Kingdom | - Walking for pleasure (including walking dog) >1 mile  - Walking to and from work > 1 mile  - Walking during work breaks > 1 mile  - Using stairs when lift is available  - Cross country hiking  - Bicycling to work and/or for pleasure  - Dancing  - Horse riding  - Home exercise  - Jogging or health club  - Running  - Weight lifting  - Sailing  - Canoeing or rowing for pleasure  - Canoeing or rowing in competition  - Swimming or surfing  - Skiing  - Ice or roller skating  - Bowling, skittles or bowls  - Cricket  - Table tennis  - Tennis or badminton  - Rugby or soccer  - Refereeing  - Squash  - Golf  - House and Garden Mowing lawn walking behind mower  - Mowing law pushing hand mower  - Weeding, cultivating, raking garden  - Spading, digging, filling in garden  - Mixing concrete by hand  - Mixing cement with machine  - Carpentry in workshop  - Painting inside of house includes paper hanging  - Carpentry outside and chopping logs  - Painting outside of house  - Fishing from river bank  - Fishing in stream with wading boots  - Hunting game, birds, rabbits | Yes | Exam | No | - - Hypertension - - Alcohol - - Cholesterol | Self-report |
| 5. | Cancer prevention study-II Nutrition Cohort  (CPS-II) | United States | - Walking   - Jogging/swimming - Lap swimming - Tennis/racquetball - Bicycling/stationary bike - Aerobics/callisthenics - Dancing - Gardening/mowing/planting - heavy home repair/painting - Shopping | No | Self-report | No | - Alcohol | Self-report |
| 6. | Doetinchem Cohort Study | The Netherlands | - Walking to work/school  - Bicycling to work/school  - Walking  - Bicycling  - Gardening  - Home repairment  - Sports | Yes | Exam | No | - - Hypertension - - Alcohol - - Cholesterol   - Family history T2D | Non fasting plasma glucose |
| 7. | Kuopio Ischaemic Heart Disease Risk Factor Study (KIHD) | Finland | - Walking on work trips - Conditioning walking - Jogging - Skiing - Bicycling - Bicycling on work trips - Swimming - Gymnastics/dancing - Ball games - Gardening/snow shovelling - Hunting/picking berries/gathering mushrooms - Fishing - Hobby crafts/repairs - Rowing - Forest work/wood cutting | Yes | Exam | Yes | - - Hypertension - - Alcohol - - Cholesterol   - Family history T2D | Self-report |
| 8. | Physical Activity Longitudinal Study  (PALS) | Canada | - Walking  - Bicycling  - Jogging/running  - Home exercise  - Exercise class/aerobics  - Ice skating  - Cross-country skiing  - Downhill skiing  - Ice hockey  - Swimming  - Gardening/yard work  - Golf  - Tennis  - Weight training  - Baseball/softball  - Popular/social dance  - Ballet/modern/jazz dance  - Square/folk dance  - Bowling | Yes | Exam | No | - - Hypertension - - Alcohol   - Family history T2D | Self-report |
| 9. | Whitehall-II study  (WH-II) | United Kingdom | - Mildly energetic activities - Moderately energetic activities - Vigorous energetic activities | No | Exam | No | - - Hypertension - - Alcohol - - Cholesterol   - Family history T2D | Plasma values |

**APPENDIX B**

**Figure B.1**. Hazard ratios and 95% CI of baseline low physical activity on development of T2D.

Model is adjusted for age, gender, educational level, smoking and BMI

**Figure B.2**. Hazard ratios and 95% CI of baseline medium physical activity on development of T2D.

Model is adjusted for age, gender, educational level, smoking and BMI

**Figure B.3**. Hazard ratios and 95% CI of baseline overweight on development of T2D.

Model is adjusted for age, gender, educational level, smoking and physical activity

**Figure B.4**. Hazard ratios and 95% CI of baseline obesity on development of T2D.

Model is adjusted for age, gender, educational level, smoking and physical activity
